# Noncoding RNAs in Nonalcoholic Fatty Liver Disease: Potential Diagnosis and Prognosis Biomarkers

**DOI:** 10.1155/2020/8822859

**Published:** 2020-08-27

**Authors:** Olfa Khalifa, Khaoula Errafii, Nayla S. Al-Akl, Abdelilah Arredouani

**Affiliations:** ^1^Diabetes Research Center, Qatar Biomedical Research Institute, Hamad Bin Khalifa University, Qatar Foundation, Doha, Qatar; ^2^College of Health and Life Sciences, Hamad Bin Khalifa University, Qatar Foundation, Education City, Doha, Qatar

## Abstract

Nonalcoholic fatty liver disease (NAFLD) is currently the most common chronic liver disease worldwide in part due to the concomitant obesity pandemic and insulin resistance (IR). It is increasingly becoming evident that NAFLD is a disease affecting numerous extrahepatic vital organs and regulatory pathways. The molecular mechanisms underlying the nonalcoholic steatosis formation are poorly understood, and little information is available on the pathways that are responsible for the progressive hepatocellular damage that follows lipid accumulation. Recently, much research has focused on the identification of the epigenetic modifications that contribute to NAFLD pathogenesis. Noncoding RNAs (ncRNAs) are one of such epigenetic factors that could be implicated in the NAFLD development and progression. In this review, we summarize the current knowledge of the genetic and epigenetic factors potentially underlying the disease. Particular emphasis will be put on the contribution of microRNAs (miRNAs), long noncoding RNAs (lncRNAs), and circular RNAs (circRNAs) to the pathophysiology of NAFLD as well as their potential use as therapeutic targets or as markers for the prediction and the progression of the disease.

## 1. Introduction

**Figure 1:**
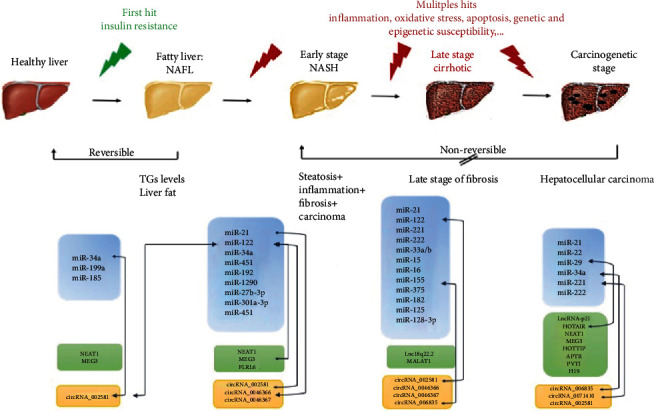
Progression of NAFLD and ncRNAs associated with each stage. The first stage of NAFLD is simple steatosis (or nonalcoholic fatty liver (NAFL)) characterized by abnormal accumulation of fat in the liver. If not reversed, simple steatosis may transform into nonalcoholic steatohepatitis (NASH), which in turn can lead to liver cirrhosis and eventually to hepatocellular carcinoma. The miRNAs, lncRNA, and circRNAs known to be associated with each stage are shown in blue, green, and yellow boxes, respectively. Few interactions between the different classes of ncRNAs in NAFLD were reported and are indicated with the arrow-ended brackets.

Nonalcoholic fatty liver disease is the accumulation of lipids in the liver above 5% of the total liver weight, in the absence of other medical conditions. It is currently the most common chronic liver disease worldwide, with a global prevalence of around 25% [1]. The high rates of NAFLD are associated with the concomitant global rise in obesity rates [2]. The pathogenesis of NAFLD implicates intricate interactions between genetic predisposition and environmental risk factors, including obesity, IR, metabolic syndrome, diabetes mellitus, and dyslipidemia [3]. NAFLD is a progressive disease and affects both adults and children. Its prevalence increases with age and is more common among males aged 45–65 years [4, 5]. The mildest form of NAFLD is steatosis (a.k.a. nonalcoholic fatty liver (NAFL)), which, if not treated, progresses to nonalcoholic steatohepatitis (NASH), an advanced stage of NAFLD in which the liver becomes inflamed, in up to 30% of patients [6]. Some 20% of individuals with NASH will progress to fibrosis, a stage characterized by the scarring of the liver [7]. In turn, around 20% of fibrosis patients will develop cirrhosis [8], which will eventually cause liver decompensation and might increase the risk of developing hepatocellular carcinoma (HCC) [9] (Figure 1). In the USA, NAFLD/NASH has become the second cause of liver transplantation [10].

The etiology of NAFLD is not well understood. However, it is widely recognized that it is closely associated with excess fat, mainly visceral adiposity, IR, T2D (type 2 diabetes), hypertension, and dyslipidemia [11]. The liver plays a crucial role in lipid metabolism, including the importing of free fatty acids (FFAs), oxidation of TGs to produce energy, synthesis of lipoproteins such as very-low-VLDL and HDL, and the conversion of excess carbohydrates and proteins into lipids. An abnormal elevation of the level of FFAs in the liver can disturb these metabolic pathways and induce IR [12]. IR is a critical factor in NAFLD pathophysiology in that it predisposes to lipolysis of peripheral fat with shunting of FFAs to the liver and exacerbates liver lipogenesis [12]. IR can also lead to liver overload with glucose, which could not be taken up by peripheral tissues, mainly skeletal muscle [13]. Through the activation of SREBP-1c and ChREBP transcription factors, the glucose induces liver synthesis of FFAs [14]. In advanced NAFLD, impairment of mitochondrial *β*-oxidation aggravates the hepatic accumulation of lipid products. This impairment is due partly to the build-up of atypical toxic lipids such as ceramides, as well as oxidative stress that develops in mitochondria and which can damage complexes of the mitochondrial respiratory chain and ultimately inhibit *β*-oxidation [15]. Besides the extrahepatic IR, it is now well known that liver IR also plays a crucial role in NAFLD and NASH [16]. The mechanisms underlying hepatic IR involve interference with the activation of the insulin receptor substrates 1 and 2 by FFA toxic metabolites like ceramides and inflammatory conditions mediated by cytokines, particularly IL-6 and TNF-*α* [15]. Furthermore, apart from the sustained uptake of glucose, there is an exacerbated hepatic gluconeogenesis, an inhibited glycogenesis, and a stimulated glycogenolysis under hepatic IR conditions [17] Together, these perturbations will increase hepatic glucose production and enhance the risk of hyperglycemia. In concert with the extrahepatic glucose, which is augmented due to peripheral IR, the glucose from hepatic origin stimulates de novo lipogenesis [18]. All these events are intertwined, and vicious cycles can arise between them.

## 2. Genetics of NAFLD

As said above, NAFLD implicates intricate interactions between many risk factors and genetic predisposition [19]. Several studies have suggested a genetic underpinning for NAFLD [20, 21]. A myriad of polymorphisms was associated with NAFLD. The SNP rs738409 C>G was the first genetic variant to be strongly related to the accumulation of fat in hepatocytes in a study that involved 2000 ethnically diverse NAFLD patients and analyzed 9229 SNPs [22]. The SNP rs738409 is located in PNPLA3 and substitutes cytosine to guanine, which changes the codon 148 of the protein from isoleucine to methionine [22]. G allele is strongly associated with NAFLD in different populations with increased risk of hepatic TG accumulation. The protein PNPLA3 has lipase activity with a role in glycerolipid hydrolysis and maximum enzymatic activity against triglycerides, diacylglycerol, and monoacylglycerol. The normal allele is C, and the worse outcome is the GG, which is associated with rapid progression to fibrosis and cirrhosis [23, 24]. The mechanism is related to the accumulation of the mutated *PNPLA3* I148M protein on the surface of lipid droplets, determining impaired FFA remodeling and reduced retinol bioavailability. The TM6SF2 (transmembrane 6 superfamily member 2) E167K variant has also been shown to increase susceptibility to progressive NAFLD [25] and is also associated with radiologically and histologically characterized NAFLD [26, 27]. TM6SF2, a gene of uncertain biological function, is located on chromosome 19 and encodes a 351 amino acid protein [25]. The TM6SF2 E167 K variant is associated with a reduction in TM6SF2 activity, which leads to an increase in liver triglyceride content by decreasing VLDL secretion [28] and enhancing the expression of some genes associated with lipid metabolism, including PNPLA3, ACSS2, DGAT1, and DGAT2 [29], and the catalytic activities of sterol isomerases [30] as well as other still unidentified molecular pathways. Carriage of the *FNDC5* rs3480 minor (G) allele was associated with more severe steatosis in NAFLD [31]. Investigations of biopsies from Caucasian women showed a strong association between SNP rs2645424 on chromosome 8 in the farnesyl diphosphate farnesyl transferase-1 gene (*FDFT-1*) and nonalcoholic steatosis (NAS) [32]. *FDFT-1* is a significant regulator gene for the biosynthesis of cholesterol. The same study reported that the level of fibrosis correlates strongly with SNP rs343062 on chromosome 7, but the exact function of this SNP is obscure [32]. SNPs rs1227756, rs6591182, and rs887304, respectively, located within the chromosome 10, 11, and 12 were associated with the lobular inflammation phenotype [32]. The SNPs rs1260326 and rs780094 in GCKR (glucokinase regulator) gene were also reported to be significantly associated with susceptibility to NAFLD and also to modulate fibrosis progression in NAFLD [33].  Table 1 shows the significant SNPs and genes related to NAFLD. A better understanding of the genetic basis of NAFLD not only will help to identify subjects at risk of NAFLD but also to dissect the pathogenesis of NAFLD and potentially develop new therapeutic strategies.

## 3. Epigenetics of NAFLD: Noncoding RNAs (ncRNAs)

Epigenetics describes the changes in gene expression caused by mechanisms unrelated to modification in the DNA sequence [34]. These mechanisms are modulated by environmental stimuli and are thus considered reversible phenomena [35]. Several disorders can result from an imbalance in these epigenetic mechanisms [34]. In response to environmental factors, the epigenetic modulation of gene expression can occur in the form of methylation of DNA nucleotides or modifications of histones that determine DNA packing and accessibility. Epigenetic modulation can also arise by regulation of transcription via alteration of stability and activity of mRNAs due to binding of specific noncoding RNAs (ncRNAs) such as microRNAs (miRNAs), circular RNAs (circRNAs), and long noncoding RNAs (lncRNAs).

Noncoding RNAs are RNAs that result mostly from alternative splicing of the more extensive transcripts, which become the precursors for smaller ncRNAs [36]. They are involved in a myriad of diseases and cellular processes, and there is also proof of their connections to create a dynamic regulatory network [37]. The ncRNAs are divided into short (<30 nucleotides), including circular (circRNAs) and microRNAs (miRNAs), and long (>200 nucleotides) ncRNAs [36]. In the below sections, we discuss the functions of the three groups of ncRNA and their potential contribution or association with NAFLD.

### 3.1. MicroRNAs (miRNAs)

The human genome encodes about 2000 miRNAs, which target 30–60% of the genes [38]. MicroRNAs are commonly deregulated in numerous diseases and are currently intensely studied, including in NAFLD [39]. Chromosome 1 encodes 134 miRs, followed by the X chromosome, which encodes 116 miRs [40]. The biogenesis of miRNAs, as well as their mechanism of action, is well established (Supplementary data 1) [41]. Numerous miRNAs are crucial regulators of liver physiological functions, including liver regeneration, lipid metabolism, apoptosis, and tissue development [42, 43]. Moreover, numerous studies have shown the dysregulation and modulation of the expression of miRNAs in NAFL, NASH, and HCC [44] (Table 2). In the section below, we take stock of a set of these miRNAs given their well-acknowledged regulatory functions in hepatic metabolism and their high therapeutic potential in fatty liver disease.

#### 3.1.1. miR-122

miR-122 is highly abundant in the liver [45]. It plays a crucial function in the epigenetic modulation of the genes linked to hepatic health. mir-122 predicted target genes include genes that regulate lipid and cholesterol metabolism [46]. Deficiency of miR-122 expression in mice leads to steatohepatitis [46]. Inhibition of miR-122 in mice reduces cholesterol levels in plasma, decreases the rates of hepatic fatty acid and cholesterol synthesis, and enhances liver fatty acid oxidation. Also, inhibition of miR-122 in diet-induced obese mice resulted in the downregulation of several lipogenic genes, decreased plasma cholesterol levels, and improved liver steatosis [46]. Further, miR122 downregulates specific genes of cholesterol biosynthesis, such as HMGCR, MTTP, and HMGCS1 [46]. Additionally, miR-122 induces the expression of de novo lipogenesis genes, including SREBP1-c, DGAT2, FAS, and ACC1, corroborating a significant physiological role of miR-122 in the biology of NAFLD. Furthermore, compared with simple steatosis, mir-122 expression is downregulated 10-fold in NASH [44], suggesting an essential role in the progression of NAFLD.

#### 3.1.2. miR-21

miR-21 is upregulated miRNAs in serum and liver from patients with fibrosing NASH and HCC [44]. Its expression was also increased in diet-induced obese mice and HepG2 cells treated with fatty acids [47]. Moreover, miR-21 knockout animals fed with a fast food diet-manifested minimal NAFL, inflammation, and apoptosis through enhanced expression of PPAR*α* and activation of FXR [48].

#### 3.1.3. miR-34a

In high-fat diet-fed mice, the levels of miR-34a were upregulated significantly in liver tissues, resulting in the downregulation of its direct hepatic targets PPAR*α* and Sirtuin 1 [49]. Furthermore, the miR-34a inhibition suppressed lipid accumulation and improved the degree of steatosis, suggesting that the downregulation of miR-34a may be a therapeutic strategy against NAFLD [50] (Table 2).

#### 3.1.4. miR-192

miR-192 is a profibrogenic implicated in the development of fibrosis and activation of TGF*β*/SMAD signaling [44]. The serum levels of miR-192 were increased by 4.4-fold in NASH patients, but in NASH, liver miR-192 is downregulated [50]. miR-192 is released from hepatocytes during pathophysiological states in humans and animal models, probably due to membrane impairment [44], suggesting its potential use as a biomarker for NASH.

#### 3.1.5. miR-370

miR-370 is a potent posttranscriptional regulator of lipid metabolism. For instance, knockdown of miR-370 in HepG2 cells results in the upregulation of lipogenic genes such as *SREBP1c* [51]. Besides, the FA oxidation enzyme CPT1A is directly targeted by miR-370. Interestingly, miR-370 may have a role in the accumulation of TGs in the liver through the modulation of miR-122 expression [51]. Further, in HepG2 cells, overexpression of miR-370 activates lipogenesis genes such as FAS and ACC1 via modulation expression of SREBP-1c [51].

#### 3.1.6. miR-33

The miR-33 family consists of two members, miR-33a and miR-33, that locate in the introns of, respectively, SREBP-2 and SREBP-1 genes [52]. mir-33 regulates lipid metabolism in the liver [53, 54]. miR-33a/b inhibits the expression of ABCA1, a major regulator of the biogenesis of HDL [54]. In mice, inhibiting the function of miR-33 raises the circulating HDL levels by targeting ABCA1 and ABCG1 [54]. miR-33 also regulates insulin signaling and reduces the oxidation of FAs. Hence, miR-33 seems to regulate both the cellular cholesterol efflux and HDL biogenesis in the liver [54].

### 3.2. Long Noncoding RNAs (lncRNAs)

lncRNAs are transcripts that cannot translate into proteins and account for most of ncRNAs [55]. They regulate transcription via gene activation or silencing through modification of chromatin [56]. The lncRNAs, because of noncomplementarity, can suppress the splicing and translation of pre-mRNA by acting as decoys of RNA-binding proteins or microRNAs [57]. They can also increase the expression of mRNAs by competing with inhibition mediated by microRNAs [57]. Interactions between lncRNA and miRNAs were also detected. Nevertheless, our understanding of miRNA-lncRNA effect on regulatory networks remains limited. The most important interaction type detected between lncRNA and miRNAs is called “Sponge effect of lncRNAs on miRNAs.” Two components are involved in this type of interaction: competing endogenous RNAs (ceRNAs) and microRNA response elements (MREs). There are currently two ways of defining the “sponge effect” of lncRNAs and miRNAs, the complete complementary mode and partial complementary mode [58]. Some of the known interactions between lncRNAs and miRNAs are shown in Figure 1.

#### 3.2.1. Biogenesis of lncRNAs

**Figure 2:**
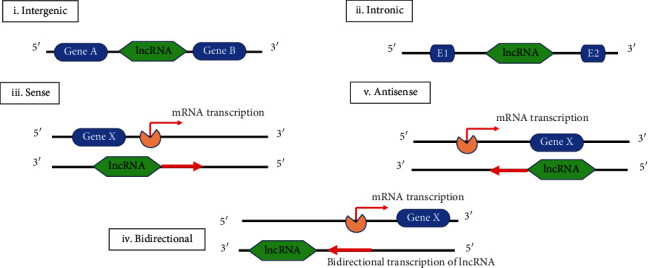
Schematic presentation of the genomic loci of different long noncoding RNA (lncRNA). lncRNA classification depends on the genomic position: (i) intergenic RNAs are located between two protein coding genes, (ii) intronic lncRNAs are positioned within an intronic region of a protein coding-gene, (iii) and (v) sense and antisense lncRNAs are transcribed from complementary strands but in different direction, respectively, and (iv) bidirectional lncRNAs originate from the bidirectional transcription of protein-coding genes.

The biogenesis of lncRNAs is not fully unraveled. Its understanding is crucial not only for distinguishing lncRNAs from other types of RNAs but also to decipher its functional significance. It is cell type- and stage-specific and is under the control of cell type- and stage-specific stimuli. To date, the molecular mechanisms underlying the lncRNAs biogenesis are not fully resolved. The lncRNA can be transcribed by the RNA polymerase II from exonic, intergenic, or the distal protein-coding regions of the genome to produce the premature lncRNA. This later gets 3′-polyadenylated and capped on the 5′-end with methyl-guanosine [59]. Epigenetic modification such as the histone methylation seems to play a key role in lncRNA biogenesis [60]. The premature lncRNAs often undergo alternative splicing to generate diverse proteins [61]. Based on the region of transcription, five types of lncRNAs can be generated: (1) bidirectional, (2) sense, (3) antisense, (4) intronic, and (5) intergenic (Figure 2). Also, small RNA deep sequencing data indicate that lncRNA could also encode small functional RNA [62]. Mature lncRNAs can be present within the nucleus and/or the cytoplasm. Although the cytoplasmic lncRNAs are not translated, small peptides that were produced from lncRNAs via their interaction with ribosomes have been identified [63]. lncRNAs can have both *cis*- and *trans*-regulatory activity [64]. As *cis*-regulators, lncRNAs affect neighbouring genes on the same allele from which they are transcribed. On the other hand, as *trans*-regulators, lncRNAs can control gene expression at a distance from their transcription site, by altering the chromatin state, influencing the nuclear structure, or regulating protein function [65].

#### 3.2.2. lncRNAs and NAFLD

Numerous lncRNAs are implicated in liver disease and have potential diagnostic, prognostic, and therapeutic importance [66]. Information about the real contribution of lncRNAs to NAFLD or its progression is scarce, though new evidence indicates that they may play an essential role in the mechanisms of the disease [66]. Although noncoding in nature, most of the lncRNAs play multiple roles in disease and biological production processes. The exact working and mode of action of lncRNA require detailed analysis. Overall, it is found that lncRNAs play a role in regulating gene expression for various diseases, including NAFLD. Four different ways of how lncRNA can work were described: signals, decoys, guides, and scaffolds [67]. In the following sections, we describe the role of the most important lncRNAs in the development and progression of NAFLD in animal models and NAFLD fibrosis patients (Table 3).

*(1) MALAT1*. This lncRNA helps cells proliferate, migrate, and invade in numerous human cancers, including HCC [68]. Knockdown of *MALAT1* expression in primary hepatic stellate cells (HSCs) from mice reduces the levels of actin alpha 2 protein (ACTA2) and *α*1 chain of type 1 collagen (COL1A1) and reduces the appearance of the myofibroblast-like morphology characteristic of activated HSCs [68]. Furthermore, palmitate-treated HepG2 and *ob/ob* mice showed that hepatic expression of *MALAT1* is enhanced compared to controls [69]. By improving the stability of nuclear SREBP-1c protein, MALAT1 also stimulates hepatic steatosis and IR [69]. Through mechanisms involving inflammatory chemokines such as *CXCL5*, a potential target for *MALAT1*, the development of fibrosis in NASH could implicate functionally relevant differences in *MALAT1* expression could. Further, knockdown of *MALAT1* expression in HepG2 cells reduces CXCL5 transcript and protein levels [70]. Also, compared to controls cells, *MALAT1* and *CXCL5* expressions enhanced inactivated liver LX-2 cells [70]. These results bring initial evidence to support a role for varying MALAT1 expression levels in the establishment of liver fibrosis in NAFLD patients (Table 3).

*(2) APTR*. APTR is a new lncRNA that regulates cell cycle progression and proliferation [71]. APTR is highly expressed in liver tissue of CCl_4_ and bile duct ligation (BDL) mice, two animal models of liver fibrosis, and in human patients with liver fibrosis [71]. The knockdown of APTR mitigated the accumulation of COL1A in vivo and suppressed the activation of HSCs in vitro [71]. Lastly, APTR levels in serum from patients with liver cirrhosis were increased, suggesting APTR as a potential biomarker for liver cirrhosis. In sum, there is data to suggest a new biological role of APTR in hepatofibrogenesis [71]. Additional studies to analyze *APTR* in sera from large cohorts will undoubtedly shed light on the implications and contribution of APTR to fibrosis attributed to NAFLD (Table 3).

*(3) MEG3*. *MEG3* is a lncRNA located in the imprinted DLK1-MEG3 locus on human chromosome 14q32.3 region [72]. Comparison of CCl_4_-treated and O-oil-fed control mice showed that *MEG3* expression was decreased in CCl_4_ mice liver, and the decrease correlated with the progression of fibrosis [73]. Similar findings were reported in fibrotic human patients [73]. In the HSC line and LX-2 cells from humans, a dose- and time-dependent downregulation of the expression of *MEG3* expression by TGFB1 was shown. In contrast, TGFB1-induced cell proliferation was inhibited and caspase-3-mediated apoptosis was promoted by the upregulation of *MEG3* in LX-2 cells [73]. Contrary to the findings in mouse models, in NASH cirrhosis and liver fibrosis in human patients, the levels of hepatic *MEG3* were significantly increased [74]. Moreover, in studies of fibrotic animals and HSCs, Yu et al. [75] identified coregulatory networks between MEG3, miR-212, and smoothened (SMO) signaling.

*(4) HOTAIR*. The expression of the lncRNA HOTAIR was reported to be upregulated in the livers of CCl_4_-treated mice and activated HSCs as compared to control counterparts [76]. On the other side, functional characterization revealed that overexpression of *HOTAIR* increases the levels of *ACTA2* and *COL1A1*, activates fibrosis-related genes, such as *MMP2* and *9 MMP9*, and promotes cell proliferation [76]. Furthermore, HOTAIR may function as an internal “sponge” of miR-148b, a regulator of the expression of the DNMT1/MEG3/p53 pathway in HSCs [76]. These findings uncover a new mechanism for epigenetic modification in liver fibrogenesis, which involves the interaction between two different lncRNAs (HOTAIR and MEG3) [76]. In NAFLD, the upregulation of HOTAIR induced by fatty acids inhibits phosphatase and tensin homolog expression gene (PTEN) and increases triglyceride accumulation in HepG2 cells [77]. Finally, aberrant upregulation of HOTAIR mediated by excessive circulating FFAs levels in the case of NAFLD may be a crucial mechanism associated with liver steatosis. In conclusion, HOTAIR may be a potential biomarker for liver injury [77].

*(5) lncRNA-COX2*. Known as prostaglandin-endoperoxide synthase 2 (*PTGS2*), cyclooxygenase 2 (*COX2*) is an enzyme involved in prostaglandin biosynthesis [78, 79] and might be implicated in liver cirrhosis [80]. Both *lncRNA-Cox2* and *Cox2* levels are enhanced in CCl_4_-treated mice as compared to controls, and the two transcripts correlated positively with the level of fibrosis [81]. These observations indicate that lncRNA-COX2 may be involved in the development of liver fibrosis and may potentially be considered a novel therapeutic target for liver fibrosis.

*(6) NEAT1*. The NEAT1 knockdown correlates with decreased proliferation, invasion, and migration of HCC cells via regulation of heterogeneous nuclear ribonucleoprotein A2 [82]. Further, in the NAFLD animal models as well as in HCC, the NEAT1 was upregulated [82]. The expression of *NEAT1* was enhanced in the livers and HSCs from CCl_4_-treated mice compared, while knockdown of *NEAT1* attenuated fibrosis in these animals [74]. By comparison, NEAT1's overexpression has facilitated HSC activation and increased levels of ACTA2 and COL1A1, indicating that this lncRNA plays a role in HSC activation.

Overexpression of NEAT1 reduces the levels of miR-122, which mediates the effects of NEAT1 effects on HSC activation, by way of a mechanism ascribed to a Kruppel-like factor 6 (Klf6) [83]. The *NEAT1*-miR-122-*Klf6* axis operates in hepatocytes and cirrhotic liver tissues from patients with unknown etiology, and the levels of *NEAT1* and *KLF6* are elevated, while those of miR-122 is reduced. Compared to controls, the levels of NEAT1 and ROCK1 were higher, and those of miR-146a-5p were lower in HepG2 cells treated by FFA and C57BL/6J mice treated by a high-fat diet [84]. On the other hand, knockdown of NEAT1 and ROCK1, and overexpression of miR-146a-5p attenuated lipid accumulation through activation of the AMPK pathway [84]. Thus, NEAT1 may regulate NAFLD through miR-146a-5p, targeting ROCK1 [84]. Another study in a NAFLD rat model reported an enhancement of the expression of NEAT1 and higher levels of ACC and FAS mRNAs [85]. Additionally, inactivation of the mTOR/S6K1 pathway had a similar effect as knockdown of NEAT1 on the expression of FAS and ACC mRNA levels [85]. Finally, the downregulated level of NEAT1 could remit the NAFLD through the mTOR/S6K1signaling pathway in rats [85].

### 3.3. Circular RNAs (circRNAs)

circRNAs are a novel class of ncRNAs containing miRNA response elements (MREs). In humans, the first endogenous circRNA was reported in 1991 [86]. Many circRNAs exist in the cell nuclei [87]. The structure of circRNA consists predominantly of a circular loop RNA void of 5′-cap and 3′-tail [88]. circRNAs are also characterized by the presence of multiple microRNA binding sites and thus function as miRNA sponges to regulate gene expression [86]. Numerous signaling cascades related to apoptosis, metastasis, vascularization, and invasion implicate the circRNA-miRNA-mRNA axes [86]. Moreover, circRNAs can regulate pathogenicity-related gene expression at the transcriptional or posttranscriptional level [86].

#### 3.3.1. Biogenesis of Circular RNAs

**Figure 3:**
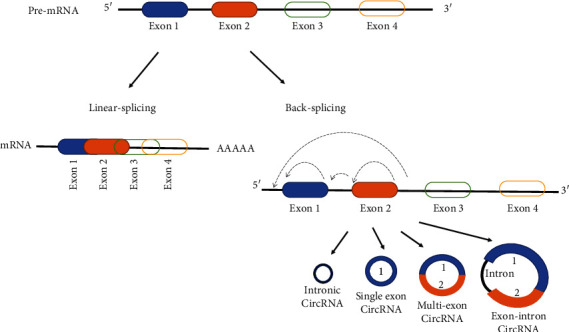
Schematic presentation of the biogenesis of circRNAs. circRNAs are lncRNAs that undergo back splicing and can originate from transcripts containing only intronic, one or more exonic, or both intronic and exonic fragments.

The biogenesis of circRNAs occurs during the transcription of most human genes due to a competition between the exonic linear splicing and an alternative splicing named back-splicing circularization [89] (Figure 3). Advances in next-generation sequencing technologies have allowed the identification of several circRNA subtypes of which four are the main ones: (1) exonic circRNAs (ecircRNAs), derived mainly from single or several exons; (2) circular intronic RNAs (ciRNAs), containing only introns; (3) exonic-intronic circRNAs (EIciRNAs), consisting of both introns and exons; and (4) tRNA intronic circRNAs (tricRNAs) are formed by splicing pre-tRNA intron. However, most of the identified circRNAs are exonic circRNAs. Furthermore, the production of different types of circRNAs is regulated by different mechanisms, which are reviewed in [89]. Spatially, ecircRNAs are mainly located in the cytoplasm, while ciRNAs, eIciRNAs, and tricRNAs are mainly distributed in the nucleus and play a crucial role in regulating parental gene transcription.

#### 3.3.2. circRNAs and NAFLD

Our knowledge about the role of circRNAs in NAFLD is scarce. Below, we review the current knowledge about the involvement of circRNAs in NAFLD progression, with a focus on the implication of the circRNA-miRNA-mRNA axis (Table 4).

*(1) circRNA_0046367 and circRNA_0046366/miR-34a/PPARα*. circRNA_0046367 and circRNA_0046366, both endogenous regulators of miR-34a, were associated with NAFLD [84]. The two circRNAs block the interaction of miRNA/mRNA with MRE and can suppress the inhibitory impact of this latter on PPAR*α* [90, 91]. In abnormal conditions, the level of PPAR*α* is increased, causing the activation of genes like carnitine CPT2 and the ACBD3, or the SLC27A, to reduce steatosis ultimately. These findings suggest that aberrant control of the signaling pathway circRNA_0046366 or circRNA_0046366/miR-34a/PPAR may be a new epigenetic mechanism underlying hepatic steatosis and provide a unique opportunity for NAFLD treatment [90].

*(2) circRNA_021412/miR-1972/LPIN1*. circRNAs profiling performed in HepG2 treated with fatty acids showed a relationship between miR-1972 and Lipin 1 (LPIN1) and confirmed the coregulation of LPIN1 expression by circRNA_021412 and miR-1972 [92]. The downregulation of the expression of long-chain acyl-CoA synthetases (ACSLs) induced by LPIN1 ultimately leads to steatosis [92]. Therefore, a decreased of circRNA_021412 levels might reduce the level of miR-1972 and might inhibit LPIN1. This circRNA-miR-mRNA signaling cascade appears to be partially involved in regulating hepatic steatosis [90].

*(3) circRNA_002581/miR-122/SLC1A5, PLP2, CPEB1*. Jin et al. [93] used liver tissues on NASH mice to perform profiling of circRNAs expression and reported that 69 and 63 circRNAs had, respectively, increased and reduced expression. Random selection of 13 from a total of 14 mRNAs and two from a total of 8 circRNAs was successfully validated by qRT-PCR. Four circRNA-miRNA-mRNA pathways were established, including circRNA_002581-miR-122-Plp2, circRNA_002581-miR-122-Cpeb1, circRNA_007585-miR-326-UCP2, and circRNA_002581-miR-122-Slc1a5 [93]. These four genes are all involved in NAFLD physiopathology.

*(4) circRNA_0067835/miR-155/FOXO3a*. Using LX 2 cells, hepatic stellate cells (HSCs) that are primary cell type responsible for liver fibrosis, Zhu et al. [94] performed a microarray test to identify thymosin beta 4 (T*β*4), a highly conserved 43 amino acid (aa) peptide that acts as an anti-inflammatory and antifibrotic agent in vitro and in vivo [95]. This study showed that circRNA_0067835, of the 644 differentially expressed circRNAs identified between control LX2 cells and the T*β*4-depleted LX-2 cells, was significantly increased in the T*β*4-depleted LX-2 cells. Bioinformatics analysis predicted that circRNA_0067835 functions as a sponge of miR-155 to regulate the expression of Forkhead Box O3 (FOXO3a) [96].

*(5) circRNA_0074410/miR-9-5p/KEGG Pathway*. circRNAs profiling of fibrotic HSCs revealed that 179 and 630 circRNAs were upregulated and downregulated, respectively. Further investigation showed that circ_0074410 reduced miR-9-5p expression and promoted HSC activation via *α*-SMA protein [97]. Moreover, inhibition of hsa_circ_0071410 upregulated the expression of miR-9-5p, leading to the attenuation of irradiation-induced HSC activation.

*(6) circRNA_34116/miR-22-3p/BMP7*. In the CCl_4_-induced mouse model of liver fibrosis, microarray screening identified 10,389 circRNAs, of which 69 were differentially expressed in the fibrotic liver tissues; 55 were downregulated while 14 circRNAs were upregulated [98]. One of the identified circRNAs is circRNA_34116. In silico analysis predicts the presence of MRE of miR-22 on circRNA_34116 and indicates that this circRNA can competitively bind to miR-22-3p and indirectly regulate the transcription of its target gene bone morphogenetic protein 7 (BMP7) [99]. To sum up, networks between circRNAs and miRNA have emerged as a new mechanism for the regulation of gene expression. They may advance our understanding of the molecular modulation of disease development and progression, and potentially open door for the discovery of novel therapeutic targets.

## 4. ncRNAs as Potential Biomarkers and Drug Targets

As discussed above, some of the reported miRNAs, lncRNAs, and circRNAs are strongly associated with NAFLD. To date, liver biopsy remains the gold standard for a firm diagnosis of NAFLD. However, the procedure remains invasive, difficult, prone to error sampling, and not practical for population screening of NAFLD. Moreover, different imaging methods have been applied to diagnose NAFLD but failed to distinguish the stages of the disease [100]. The implication of the regulatory networks of circRNAs, lncRNAs, and miRNAs in the pathophysiology of NAFLD offers new opportunities in identifying novel diagnostic and prognostic biomarkers as well as therapeutic targets. Establishment of sensitive and noninvasive biomarkers for NASH and fibrosis is paramount given the invasiveness of the biopsy, which can further complicate liver health and the limits of the imaging methods, which cannot detect NASH [101]. NAFLD-associated circulating miRNAs were proposed as potential noninvasive markers to be used in clinical practice. They may be more specific and sensitive biomarkers for fatty liver disease, disease stage and disease diagnosis, and drug targets. These kinds of miRNAs have been used not only in the experimental research but also used clinically for early detection and prevention of cancer progression [102]. Circulating miRNAs are stable and resistant to the degradation by ribonucleases and easily detected in the peripheral circulation [103, 104]. Some of the circulating miRNAs are abnormally expressed in NAFLD patients [105]. Tan et al. [105] reported an upregulation of miR-122, miR-27b-3p, miR-192, and miR-148a-3p. The levels of miRNA-122 were increased not only in the human liver but also in the blood circulation in NAFLD patients [106, 107]. miR-122 is identified in other liver diseases such as viral hepatitis B and C [108, 109]. Further, levels of circulating miR-122 and miR-34a are correlated positively with the clinical traits and the stage of progression of the disease [110]. Similar to miRNAs, some lncRNAs were also observed in NAFLD, increasing evidence that characterize lncRNAs role in NAFLD pathogenesis and several functions such as lipogenesis, insulin resistance, and fatty acid oxidation. In vivo animal models and liver tissue studies revealed an association of MEG3, APTR, MALAT1, PVT, SRA, HOTAIR, NEAT1, and others to the development and progression of NAFLD. There is considerable interest in their potential use as biomarkers or therapeutic targets [111]. However, further investigations are required to fully unravel their functional targets as well as their secretion into the circulation.

As we discussed above, miRNAs are becoming potential noninvasive biomarkers for the diagnosis, prognosis, and therapeutic targets of several diseases. Despite its many benefits, there are still obstacles and challenges to be surmounted before their adoption in clinical applications. First, the fundamental technical constraint to solve is the isolation and purification of samples, as the quality and purity of RNA is the basis of detection and quantification. Unlike intercellular miRNAs, circulating miRNAs are easily interfered by other serum components, and one need to be vigilant when purifying from serum [102]. Second, one of the most critical aspects of the ultimate results of circulating miRNAs is the source of the samples. The expression of miRNAs is different even in the same person among the samples extracted from the serum and plasma [112]. Third, and despite the availability of sensitive quantifiable detection methods of circulating miRNAs such as quantitative PCR, microarray, and next-generation sequencing, it is still hard to measure circulating miRNAs accurately because of their often-low concentration.

Many lncRNAs play significant roles in multiple physiological processes involving gene regulation, as mentioned above, but it also opens the possibility of using these types of RNAs as diagnostic markers and therapeutic targets particularly when they can be readily detected in biological fluids [113]. Furthermore, like miRNAs, the lncRNAs are a functional molecule and they can be a better indicator of the disease. For the moment, lncRNAs could be successfully used for accurate disease diagnostics [113]. Nevertheless, the evolving application of circulating lncRNAs for diagnosis of the disease is restricted by the limited knowledge that we have of their biology. Some challenges were present in the case of miRNAs use for diagnosis. For example, it is unknown whether lncRNAs contribute to the disease or whether they deregulate as a consequence of the disease itself. Besides, given their existence as long RNA molecules, are lncRNAs stable in circulation? Is their stability changed in various disease states? Solving those inquiries will help to apply miRNAs and lncRNAs as biomarkers. To date, the liver biopsy remains the gold standard for the diagnosis of NAFLD stages. However, as current techniques evolve, it is anticipated that lncRNAs will become a routine approach in the development of personalized patient profiles, thus permitting more specific diagnostic, prognostic, and therapeutic interventions.

Given the high stability of circRNAs in circulation, there is considerable interest in their potential use as biomarkers or therapeutic targets [114]. Unfortunately, most studies on circRNAs performed in liver diseases focused on HCC and hepatitis [92]. There is a need for further investigations to unravel the mechanisms of how these ncRNAs modulate the progression of NAFLD and identify which molecules they interact with, notably in regard to lncRNAs that have diverse biological functions.

## 5. Conclusion and Perspectives

The development of several diseases, including NAFLD, involves numerous genetic and epigenetic factors. With the advance in high-throughput profiling methods, the coming years will undoubtedly see the discovery of new genetic determinants of NAFLD. Moreover, the interaction of epigenetic changes with inherited risk factors to determine an individual's susceptibility to NAFLD will require more investigations to unravel the underlying mechanism fully. So far, no therapy exists for NAFLD, and the lifestyle modification aimed at weight loss remains the only therapy that gave relatively promising results. The evaluation of circulating ncRNA represents a promising strategy to assess and noninvasively monitor liver disease severity. Still, more investigations are required to identify and validate the efficiency and accuracy of these markers and to study their therapeutic potential. In a nutshell, studying the ncRNA in NAFLD will shed light on the pathophysiology of the disease. Still, it can also potentially help identify novel drug candidates as well as noninvasive and accurate predictive, diagnostic, and prognostic biomarkers.

## Figures and Tables

**Table 1:** Candidate genes and variant associated with NAFLD: function and phenotype.

Pathway	Genes	Variant/SNP	Phenotype/function	Stage/phase	Studies	References
Lipid metabolism	PNPLA3	rs738409 C/G	Hepatic steatosis, histologic lobular inflammation, HCC fibrosis, lipolytic and lipogenic function in vitro	NAFLD/NASH/activated HSCs	GWAS, case-control	[22, 115],
	PPAR*γ*	rs1801282 G/C	Protection against liver injury	NAFLD	Case-control, GWAS, meta-analysis	[116]
	LPIN1	rs13412852 C/T	Regulation of lipid metabolism, reduced lipolysis, decreased flux of FAs to the liver, decreased fibrosis	NAFLD	Case-control	[115]
	NCAN	rs2228603 C/T	Regulates cell adhesion and migration/hepatic steatosis	NAFLD	GWAS	[115]
	*RETN*	rs3745367 G/A	Involved in lipid metabolism, hepatic insulin resistance, inflammatory cascade reactions, and fibrogenesis	NAFLD	GWAS, case-control	[115]

Cholesterol biogenesis	SREBPF 1	rs11868035 A/G	The severity of steatosis and necroinflammation/impaired glucose homeostasis and lipoprotein and adiponectin responses to fat ingestion	NAFLD NASH	Case-control	[117]
	SREBPF 2	rs2228314 G>C	Histological characteristics and NASH diagnosis	NASH	Case-control	[118]

Fatty acid uptake and transport	PPAR	rs1801282	Protection against liver injury	NAFLD	Case-control, GWAS, meta-analysis	[119]
	APOC3	rs2854116 T/C rs2854117 C/T	Hepatic steatosis/inhibits lipoprotein lipase and triglyceride clearance	NAFLD	Case-control, meta-analysis	[120]
	FABP1	rs2241883 T/C rs1545224G/A	Impact blood lipoprotein/lipid levels and responses to lipid-lowering therapy and glycogenolysis/fibrosis steatosis	NAFLD/NASH	Case-control	[121]
	MTTP	rs1800591 rs1800804 rs1057613	Synthesis and secretion of VLDL in the liver, transfer protein involved in apoB-lipoprotein assembly/hypobetalipoproteinemia Fibrosis, steatosis, and increased histological grade of NASH	NAFLD/NASH	GWAS, case-control	[120]

Oxidative stress	PPAR*α*	rs1800206	Steatosis, inflammation, and fibrosis Activates fatty acid oxidation and hepatic lipid hydrolysis	NAFLD/NASH	GWAS, case-control	[121]
	PNPLA3	rs738409 C/G	Hepatic steatosis, histologic lobular inflammation, HCC development, fibrosis/lipolytic and lipogenic function in vitro	NAFLD/NASH/activated HSCs	GWAS, case-control	[120]
	TM6SF2	rs58542926	Hepatic steatosis, necroinflammation, ballooning, higher serum alanine aminotransferase (ALT) and aspartate aminotransferase (AST) levels/fibrosis, cirrhosis	NAFLD/NASH	GWAS	[22, 115],
	GCKR	rs780094 A>G rs1260326 C>T	Steatosis/fibrosis, inability to regulate glucose influx into hepatocytes, increased de novo lipogenesis	NAFLD/NASH	GWAS, meta-analysis	[20, 27],
	HSD17B13	rs72613567 T>A	Localizes to hepatocyte lipid droplets/decreased HSD17B13 and PNPLA3 production	↓NASH ↓Fibrosis	Case-control	[20]

Insulin resistance	ADIPOQ	rs2241766 rs1501299	Insulin-sensitizing, anti-inflammatory adipokine/severity of liver disease and with an atherogenic postprandial lipoprotein profile in NASH	NAFLD	Case-control	[115]
	IRS1	rs1801278	Downstream regulator of insulin action, deceased insulin signaling/fibrosis	NAFLD	Case-control	[115]
	PPAR*γ* C1*α*	rs8192678	Transcriptional coactivator that regulates genes involved in lipid and glucose metabolism	NAFLD	Case-control	[122]
	TCF7L2	rs7903146C/T	Regulates gene expression in cellular metabolism and growth	NAFLD	Case-control	[122]

**Table 2:** Specific miRNAs with NAFLD development and progression in human patients.

Candidate biomarkers	Human		Stage/phase	Function	Approach	References
	Serum	Liver				
miR-122	Upregulated	Downregulated	NAFLD/NASH/activated HSCs	Lipid metabolism, intestinal permeability, inflammation, fibrogenesis, proliferation	RT-qPCR Illumina sequencing, miRNA PCR-based array	[123]
miR-192	Upregulated	Downregulated	NAFLD/NASH/activated HSCs/fibrosis	HSC activation	RT-qPCR miRNAs PCR-based array	[123]
miR-34a	Upregulated	Upregulated	NAFLD/NASH/steatosis/activated HSCs/fibrosis	Inflammation	RT-qPCR miRNAs PCR-based array	[123]
miR-1290	Upregulated	Upregulated	NASH	Inflammation	RT-qPCR Illumina sequencing	[105]
miR-27b-3p	Upregulated	Upregulated	NASH	Inflammation	RT-qPCR Illumina sequencing	[123]
miR-192-5p	Upregulated	Upregulated	NASH	Inflammation	RT-qPCR Illumina sequencing	[123]
miR-34a-5p	Upregulated	Upregulated	NASH/NAFLD	Lipid metabolism, oxidative stress, apoptosis, useful miRNA biomarkers to discriminate between NAFLD and NASH patients	RT-qPCR	[44]
miR-375	Upregulated	Upregulated	NAFLD/NASH/fibrosis	Glucose homeostasis, intestinal permeability modulation, inflammation	RT-qPCR miRNAs PCR-based array	[50]
miR-155	Upregulated	Upregulated	NASH	Master regulator of inflammation	RT-qPCR	[124]
miR-125b	Upregulated	Upregulated	Steatosis/NASH/fibrosis	Lipid and glucose homeostasis, adipocyte differentiation, fibrogenesis	RT-qPCR	[50]
miR-33a/b	Upregulated	Upregulated	NASH	Lipid and cholesterol metabolism, glucose homeostasis	RT-qPCR	[125]
miR-451	Upregulated	Downregulated	NASH/NAFLD	Inflammation	RT-qPCR	[126]
miR-155	Upregulated	Upregulated	NASH	Lipid metabolism, intestinal permeability modulation, inflammation	RT-qPCR	[127]
miR-221	Upregulated	Upregulated	NASH/fibrosis/HCC	HSC activation, fibrogenesis	RT-qPCR	[128]
miR-222	Upregulated	Upregulated	NASH/fibrosis/HCC	HSC activation, fibrogenesis	RT-qPCR	[128]
miR-15	Upregulated	Downregulated	Fibrosis/HCC	HSC activation, proliferation, and metastasis	RT-qPCR	[129]
miR-16	Upregulated	Downregulated	Fibrosis/HCC	HSC activation, proliferation, and metastasis	RT-qPCR	[123]
miR-21	Upregulated	Upregulated	NASH/fibrosis/HCC	Gut microbiota modulation, inflammation, proliferation	RT-qPCR	[130]
miR-22	Upregulated	Upregulated	NASH/fibrosis/HCC	Inflammation	RT-qPCR	[123]
miR-29	Upregulated	Upregulated	NAFLD/NASH/activated HSCs	Inflammation	RT-qPCR	[131]

**Table 3:** Confirmed lncRNAs in NAFLD.

Name of lncRNA	Type of lncRNA	Chromosome	Expression	Stage	Gene targets	References
MEG3	Intergenic	14q32	Downregulated in human hepatic	NAFLD/NASH Activated HSCs	*DNMT* and *TGFB1*	[72, 73]
MALAT-1	Intergenic	11q13.1	Upregulated Identified in animal models MALAT1 knockdown leads to downregulation of CXCL5 Plays a key role in tumor cell proliferation, migration, and invasion	Fibrosis	*CXCL5*	[132]
HOTAIR	Antisense	12q13.13	Upregulated/replicated in human	HCC	*PTEN*	[133]
APTR	Intergenic	7q21	Upregulated/involved in regulating cell cycle progression and cell proliferation in liver cirrhosis	HCC	*TGF-β1*	[134]
PVT1	Intergenic	8q24.21	Upregulated/not replicate in human	HCC	*miR-152*	[135]
lnRNA-CoX2	Antisense	1p33	Upregulated	Liver fibrosis HCC	*PTGS2*	[80, 81],
NEAT1	Intergenic	11q13.1	Upregulated	NAFLD/NASH Activated HSCs	*ACTA2* and *Col1a1*	[83]

**Table 4:** circRNA in NAFLD.

Name of circRNA	Target miRNA	Gene targets	Expression	Stage/phase	References
*circRNA_34116*	*miR-22-3p*	*BMP7*	Upregulated	HCC	[98]
*circRNA_0074410*	*miR-9-5p*	*KEGG pathway*	Downregulated	NAFLD	[97]
*circRNA_0067835*	*miR-155*	*FOXO3a*	Upregulated	NAFLD	[94, 95],
*circRNA_002581*	*miR-122*	*SLC1A5*, *PLP2*, *CPEB1*	Upregulated	NAFLD	[93]
*circRNA_0046367* and *circRNA_0046366*	*miR-34a*	*PPARα*	Upregulated Upregulated	NAFLD NAFLD	[84, 91],
*circRNA_021412*	*miR-1972*	*LPIN1*	Upregulated	NAFLD	[136]

## Abbreviations

ACBD3: Acyl-CoA-binding domain-containing 3
ACC: Acetyl-CoA carboxylase
ACSS2: Acyl-CoA synthetase short-chain family member 2
ACTA2: Actin alpha 2
APTR: Alu-mediated transcriptional regulator
ChREBP: Carbohydrate-responsive element-binding protein
circRNA: Circular RNA
COL1A1: Chain of type 1 collagen
CXCL5: C-X-C motif chemokine ligand 5
DGAT1: Diacylglycerol O-acyltransferase 1
DGAT2: Diacylglycerol O-acyltransferase 2
DNMT1: ADN-methyltransferase 1
FDFT-1: Farnesyl diphosphate farnesyl transferase-1 gene
FFAs: Fatty free acids
FNDC5: Fibronectin domain-containing protein 5
FXR: Farnesoid X-activated receptor
GWAS: Genome-wide association scan
HCC: Hepatocellular carcinoma
HDL: High-density lipoprotein
HMGCS1: Hydroxy methylglutaryl-coenzyme A synthase 1
HOTAIR: Homeobox transcript antisense RNA
IL-6: Interleukin-6
IR: Insulin resistance
lncRNA-COX2: lncRNA-cyclooxygenase 2
lncRNA: Noncoding RNAs
LPIN1: Lipin 1
MALAT1: Metastasis-associated lung adenocarcinoma transcript 1
MEG3: Maternally expressed gene 3
miRNA: MicroRNA
MMP2: Matrix metalloproteinase 2
MMP9: Matrix metalloproteinase 9
mRNA: Messenger RNA
MTTP: Microsomal TG transfer protein
NAFL: Nonalcoholic fatty liver
NAFLD: Nonalcoholic fatty liver disease
NAS: Nonalcoholic steatosis
NASH: Nonalcoholic steatohepatitis
ncRNAs: Noncoding RNAs
NEAT1: Nuclear-enriched abundant transcript 1
PNPLA3: Patatin-like phospholipase domain-containing protein 3
PPAR*α*: Peroxisome proliferator-activated receptors
PTEN: Tensin homolog expression
PVT1: Plasmacytoma variant translocation 1
SNP: Single-nucleotide polymorphism
SREBP-1c: Sterol regulatory element-binding transcription factor 1
TM6SF2: Transmembrane 6 superfamily members 2
VLDL: Very-low-density lipoprotein.

## Supplementary Materials

Supplementary Materials Supplementary data 1: overview of the miRNA biogenesis pathway. miRNA genes are transcribed in the nucleus by RNA Pol II as long pri-miRNA transcripts that are 5′ capped and 3′ polyadenylated. The pri-miRNA processed by the microprocessor complex Drosha-DGCR8, generating a pre-miRNA. The pre-miRNA is exported from the nucleus to the cytoplasm by exportin 5, where it is further cropped by Dicer in complex with TRBP, yielding a ~22 nt double-stranded RNA called miRNA/miRNA∗ duplex. The functional mature miRNA is loaded together with Argonaute proteins into the RISC complex, guiding RISC to silence a target mRNA through translational repression or deadenylation.
