# Supplementary material for: Noncoding RNAs in Nonalcoholic Fatty Liver Disease: Potential Diagnosis and Prognosis Biomarkers
Source: Dis Markers. 2020 Aug 27;2020:8822859. doi: 10.1155/2020/8822859 (PMC7593715; doi:10.1155/2020/8822859)
Supplement: Supplementary Materials — Supplementary data 1: overview of the miRNA biogenesis pathway. miRNA genes are transcribed in the nucleus by RNA Pol II as long pri-miRNA transcripts that are 5′ capped and 3′ polyadenylated. The pri-miRNA processed by the microprocessor complex Drosha-DGCR8, generating a pre-miRNA. The pre-miRNA is exported from the nucleus to the cytoplasm by exportin 5, where it is further cropped by Dicer in complex with TRBP, yielding a ~22 nt double-stranded RNA called miRNA/miRNA∗ duplex. The functional mature miRNA is loaded together with Argonaute proteins into the RISC complex, guiding RISC to silence a target mRNA through translational repression or deadenylation. [file 8822859.f1.docx]

**Nucleus**

**Cytoplasm**

**DNA polymerase II**

**Transcription**

**Exportin-5**


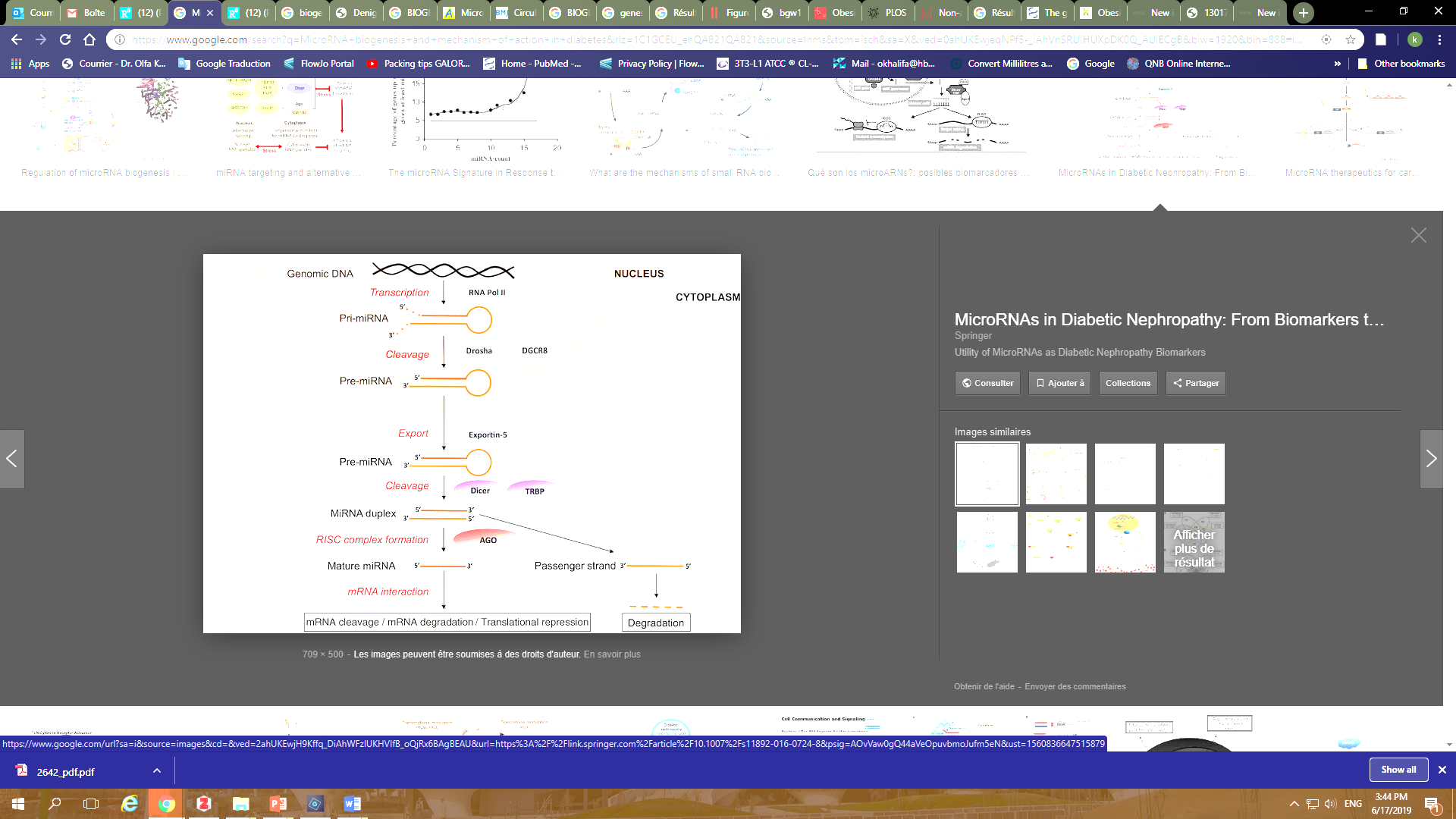


**AAA**

**Exporting**

**microRNA gene**

***Pri-miRNA***

**AAA**

***miRNA duplex***


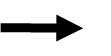


**Cropping**

***Pre-miRNA***


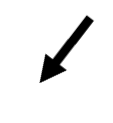

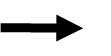

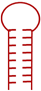


**Cleavage**


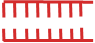


**3’**

**5’**

**3’**

**5’**


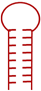


***Pre-miRNA***

***Mature miRNA***


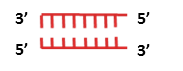


**Degradation**


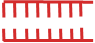


**3’**

**5’**


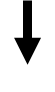


**RISC-complex formation**


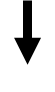

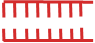


**3’**

**5’**

**3’**

**5’**


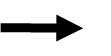


**mRNA target cleavage**

**Translational repression**

**mRNA deadenylation**


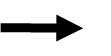

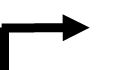

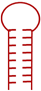

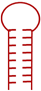

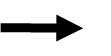

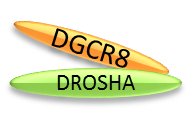

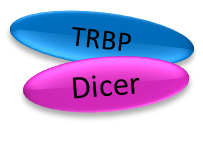


**Supplementary data 1. Overview of the miRNA biogenesis pathway**. miRNA genes are transcribed in the nucleus by RNA Pol II as long pri-miRNA transcripts that are 5 ′ capped and 3 ′ polyadenylated. The pri-miRNA processed by the Microprocessor complex Drosha-DGCR8, generating a pre-miRNA. The pre-miRNA is exported from the nucleus to the cytoplasm by exportin 5, where is further cropped by Dicer in complex with TRBP, yielding a ~22 nt double-stranded RNA called miRNA/miRNA* duplex. The functional mature miRNA is loaded together with Argonaute proteins into the RISC complex, guiding RISC to silence a target mRNA through translational repression or deadenylation.
